# Protocol for the establishment of the Pediatric Registry for Stroke as a Multidisciplinary Approach to healthcare research (PRiSMA) study

**DOI:** 10.1371/journal.pone.0341646

**Published:** 2026-03-03

**Authors:** Lucia Gerstl, Victoria Lieftüchter, Eva Jung, Florian Heinen, Dewi Bakker, Steffen Berweck, Mark Dzietko, Thomas Liebig, Martin Olivieri, Rainer Seidl, Ronald Sträter, Clara Haacke, Barbara Lippert, Sarah Glatter, Jakub Fusiak, Jonathan Christ, Michael Lauseker, PRiSMA study group

**Affiliations:** 1 Division of Pediatric Neurology and Developmental Medicine, Department of Pediatrics, Dr. von Hauner Children’s Hospital, Ludwig-Maximilians-University (LMU) Hospital, LMU Munich, Munich, Germany; 2 Munich University Center for Children with Medical and Developmental Complexity, LMU Hospital, LMU Munich, Munich, Germany; 3 Division of Pediatric Intensive and Emergency Care, Department of Pediatrics, Dr. von Hauner Children’s Hospital, Ludwig-Maximilians-University (LMU) Hospital, LMU Munich, Munich, Germany; 4 Department of Neuropediatrics and Muscle Disorders, Faculty of Medicine, Medical Center, University of Freiburg, Freiburg, Germany; 5 Pediatrics, Department of Pediatric Neurology, Amsterdam University Medical Center, Amsterdam, the Netherlands; 6 Department of Child Neurology, Developmental Medicine and Rehabilitation, Children’s Hospital of Eastern Switzerland, St. Gallen, Switzerland; 7 Department of General Pediatrics, Neonatology and Pediatric Cardiology, Medical Faculty, University Hospital Düsseldorf, Heinrich-Heine-University, Düsseldorf, Germany; 8 Institute of Neuroradiology, Ludwig-Maximilians-University (LMU) Hospital, LMU Munich, Munich, Germany; 9 Paediatric Thrombosis and Haemostasis Unit, Paediatric Haemophilia Centre, Dr. Von Hauner Children´s Hospital, Ludwig-Maximilians-University (LMU) Hospital, LMU Munich, Munich, Germany; 10 Department of Pediatrics and Adolescent Medicine, Medical University Vienna, Vienna, Austria; 11 Department of General Pediatrics (Neuropediatrics), University Hospital Muenster, Muenster, Germany; 12 Institute for Medical Information Processing, Biometry, and Epidemiology, Faculty of Medicine, Ludwig-Maximilians-Universität, Munich, Germany; PLOS: Public Library of Science, UNITED KINGDOM OF GREAT BRITAIN AND NORTHERN IRELAND

## Abstract

**Background:**

Childhood stroke is a rare but potentially life-threatening event that can occur at any age. It requires prompt diagnosis and appropriate treatment, and, in view of the frequent neurological and neurocognitive sequelae, long-term care in a multidisciplinary and multiprofessional setting. The management of stroke in children, which differs in many aspects from stroke in adults, often relies on local expert opinions and retrospectively collected data rather than high-quality evidence from prospective randomized controlled clinical trials, especially in the acute phase.

**Aims:**

The **Pediatric Registry for Stroke as a Multidisciplinary Approach to healthcare research (PRiSMA)** study aims to systematically collect longitudinal prospective observational data across the acute and long-term phases to optimise diagnosis, personalised acute therapy, neurorehabilitation, and prevention of recurrent stroke. The data will inform development of further hypothesis-driven studies of childhood stroke in national and international networks.

Furthermore, PRiSMA will facilitate case-specific collaboration within the registry network, allowing treating clinicians to consult experienced colleagues on complex cases.

**Methods:**

In PRiSMA all children and adolescents (>28 days of life, ≤ 18years) with arterial ischemic stroke (AIS) or hemorrhagic stroke (HS) are eligible for recruitment. The nationwide multicenter, systematic data collection is performed in the acute and chronic phases, extracted from patient medical records supplemented by direct registry-specific medical history provided by caregivers. In addition, a questionnaire-based data on quality of life and behaviour will be collected. International collaborations are established across Germany, Austria and the Netherlands.

**Status:**

Currently, 16 centres in Germany and 1 centre in Austria have commenced recruitment. 30 patients are currently (17 Nov 25) enrolled after arterial ischaemic stroke and 1 after haemorrhagic stroke.

## Introduction

Paediatric stroke can occur at any age and is one of the most time-critical emergencies in paediatric and adolescent medicine. Many of those affected have long-term neurological impairments, such as hemiparesis and epilepsy [1,2]. However, the neurocognitive effects on behaviour and well-being should not be underestimated, as they have a significant impact on a child’s ability to engage in normal activities [3–5].

Evidence-based diagnostic algorithms and treatment recommendations are available for adult stroke where new models of care, such as neurovascular networks, have been successful, and stroke units have been established throughout Germany. However, these extensive, dynamic and positive experiences in adult neurology cannot simply be transferred to the paediatric population due to the specificities of a child’s developing brain and the fundamentally different aetiologies of stroke in children [6–8].

The approach to paediatric stroke, as a rare disease, is often empirical and based on the expert opinions and retrospectively collected observational data. Furthermore, prospective clinical trials, particularly in the acute phase of paediatric stroke, are often not thought feasible [9,10].

In Germany there is a paucity of comprehensive scientific analysis with systematic assessment of patient data in the acute and long-term phases, which could form the basis for optimised diagnostics, personalised acute therapy, recurrence prevention and neurorehabilitation. This underscores the need for systematic, prospective data collection to improve diagnostics and outcomes. We therefore need reliable data that also includes follow-up information on these patients and their treatment.

## Materials and methods

### Aims

The primary aim of the multicentre PRiSMA study is to prospectively collect longitudinal data that will enable a broad range of research questions to be addressed in the fields of epidemiology, healthcare provision, therapy, and prognosis. These questions include, but are not limited to, identifying prognostic and risk factors for paediatric stroke; evaluating quality of life over the course of the disease; comparing the application of different acute and secondary preventive therapies nationally and across Europe; and describing the quality and speed of initial diagnosis and acute care. The findings from the collected data will translate to improvements in acute and long-term care, leading to the adaptation and further development of childhood stroke guidelines.

The collected data will also support subsequent hypothesis-driven studies aimed at identifying children at particularly high riskof stroke and ensure they receive earlier, personalised intervention. Another important aim of PRiSMA is to build a consultation network. Modern medicine increasingly relies on interdisciplinary networking. In everyday clinical practice, many paediatricians only see and treat a few children with strokes, who may differ from each other in terms of clinical presentation, aetiology, course and treatment requirements. PRiSMA will facilitate professional exchange between paediatric neurologists, thus contributing directly to improving medical care for children with this rare disease.

Lastly, planned awareness campaigns targeting caregivers and emergency department physicians are intended to improve care for children by enabling earlier stroke diagnosis.

### Procedure

The PRiSMA paediatric stroke registry is a multicentric, observational cohort study. So far 17 hospitals treating paediatric stroke patients are participating and enrolling patients. Further 6 centres are in the process of contract and ethics approval. In the first step, the study covers Germany as well as Austria and the Netherlands. Further European and international collaborations are planned.

Patients are recruited to the registry by the treating clinician at each study centre who obtains signed informed consent. Clear, age-appropriate informational materials are provided to the patient and their parents or legal representatives. Enrolment in the registry occurs only after obtaining a written informed consent form from the patient and their legal guardians. In accordance with data protection laws, a patient can withdraw his or her consent at any time.

If the patient and parents agree, they may be contacted by the coordinating study centre in Munich for a questionnaire-based survey on quality of life (QoL) and behaviour. A separate written informed consent is required for this component of PRiSMA.

### Study population

Internationally, the incidence of stroke in children is reported to be 1–8 per 100,000 children [11,12], but reliable data for Germany are lacking. Based on data from the German Federal Statistical Office, around 14 million people under 18 years live in Germany [13], which would expect 140–1120 cases of paediatric stroke per year. Considering the findings from the nationwide, multicentred ESPED study [1] into account, we would include approximately 55 patients with ischemic stroke annually, along with those newly diagnosed with haemorrhagic stroke. Including the patients from Austria and the Netherlands, approximately 100 patients per year can be expected to be enrolled. As an extension to other European countries is intended, the number should further increase in the future.

### Inclusion and exclusion criteria

Prospectively included are all children and adolescents (>28 days to 18 years of age) with radiologically confirmed arterial ischemic (AIS) or haemorrhagic stroke (HS) (first event or recurrence).

Exclusion criteria are acute symptomatic neonatal stroke, suspected perinatal stroke diagnosed beyond the neonatal period, traumatic brain haemorrhage, and infarction (haemorrhagic or ischemic) secondary to sinus or cerebral vein thrombosis. In the absence of consent, inclusion is not possible.

### Data collection

1 **Medical data**

The data collected are observational, relying primarily on medical records as the source of information. Data are collected prospectively, with the exception of information with regard to stroke risk factors prior to the index event, which have to be collected retrospectively.

**Fig 1:**
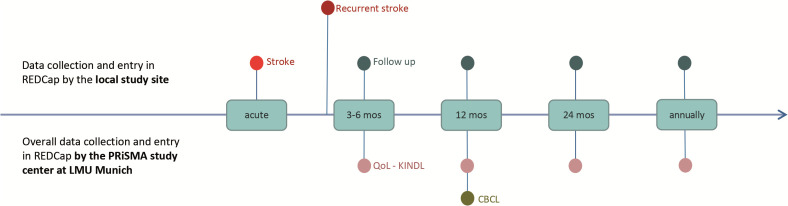
Time points of data collection. **CBCL** – Child Behavior Checklist, **KINDL** – quality of life questionnaire, **QoL** – Quality of Life.

For each patient, we are interested in timelines of care for the index or recurrent stroke event. Additionally, follow-up information is collected at three to six months and twelve months after stroke, and then annually until the patient reaches adulthood. These intervals correspond to the usual clinical follow-up appointments (see Fig 1).

Data collection will be aligned with routine clinical appointments, to minimize research burden on families. If there is no doctor-patient contact on any of the proposed dates, no entry can be made in the registry. Data is entered into the database directly by the centres. An overview of collected data for both AIS or HS is shown in the Table 1.

**Table 1:** Overview of collected data of children with arterial ischemic stroke (AIS) or haemorrhagic stroke (HS) (categories, further details available on request).

CRF		
	**Data category**	**Details on**
**Acute**	Demographics	Age, sex, ethnicity, residence degree of urbanisation
	History of stroke event	Time of onset Clinical symptoms
	Medical history and previous development	Pre-existing conditions Development normal/with concerns
	Family history	Risk factors for stroke, cardiovascular/ thrombotic/ bleeding events in family members < 50 years of age
	Clinical examination at admission	GCS AWUP PedNIHSS Vital parameters (HR BP, temperature, ….)
	Neuroimaging (AIS)	Time of neuroimaging Modality (cCT, cMRI, cMRA,…) Results (incl. localization, affected vessel, results of vesselwall imaging, DWI/FLAIR mismatch, etc)
	Neuroimaging (HS)	Time of neuroimaging Modality (cCT, cMRI, cMRA,…) Bleeding volume (IVH: Graeb Score, SAH: Fisher Scale) Further specification: Aneurysm, AVM (Spetzler-Martin scale), cavernoma, dissection, angiopathy,…
	Diagnostic work up	Neurophysiology (i.e., EEG…) Cardiac work up (ECG, TTE, TEE) Laboratory testing (BC, vasculitis, coagulation, infectious, genetic testing)
	Acute Treatment (AIS)	Time of intervention Recanalization (thrombolysis, thrombectomy), antithrombotic treatment, immunosuppression etc.
	Acute Treatment (HS)	Time of intervention Interventional neuroradiology Neurosurgery
	Clinical course	Complications (arterial hypertension, seizures, increased intracranial pressure, infection)
	time of discharge clinical symptoms Treatment Follow up	Neurological examination, mRS Antithrombotic treatment, immunosuppression, ASM, other rehabilitation
	Stroke classification (AIS)	CASCADE criteria
	Stroke classification (HS)	ICH, IVH, SAH i.e., AVM, cavernoma, aneurysm, tumor, hypertension, medication induced, coagulation disorder, other
**Follow-up**	Demographics	Age at follow up
	Full recovery after stroke? (subjective)	Yes/no (further specification)
	childcare, school	Special educational needs?
	Aspects of social law	Level of care, Severely disabled pass
	Transition (for adolescent patients)	
	Family history	Any new aspects not reported above?
	Intermittent diagnostics	Neuroimaging, laboratory/genetic testing, cardiac test, neurophysiology, neuropsychology,…
	Medication	Antithrombotic treatment Immunosuppressive treatment Antiseizure medication other
	Functional therapies	i.e., speech therapy, physiotherapy, OT, psychotherapy
	Intermittent interventions/surgery	i.e., interventional neuroradiology, neurosurgery, cardiac surgery
	Neurological examination	Neurological sequelae mRS GMFCS, MACS, BFMF, CFCS,…
	Stroke classification	Any change in diagnosis?
**Recurrent stroke**	Demographics	Age at stroke recurrence
	History of stroke	Time of Onset Clinical symptoms
	Clinical examination at admission	GCS AVUP PedNIHSS Vital parameters (HR, BP)
	Neuroimaging (AIS)	Time of neuroimaging Modality (ccT, cMRI, cMRA,…) Results (incl. localization, affected vessel, results of vesselwall imaging, DWI/FLAIR mismatch, etc)
	Neuroimaging (HS)	Time of neuroimaging Modality (ccT, cMRI, cMRA,…) Bleeding volume (IVH: Graeb Score, SAH: Fisher Scale) Further specification: Aneurysm, AVM (Spetzler-Martin scale), cavernoma, dissection, angiopathy,…
	Current medication	Antithrombitic treatment Immunosuppressive treatment Antiseizure medication other
	Acute Treatment (AIS)	Time of intervention Recanalization (thrombolysis, thrombectomy), antithrombotic treatment, immunosuppression etc
	Acute Treatment (HS)	Time of intervention Interventional neuroradiology neurosurgery
	Clinical course	Complications (arterial hypertension, seizures, increased intracranial pressure, infection,…)
	time of discharge - clinical symptoms - Treatment	Neurological examination, mRS Antithrombotic treatment, immunosuppression, ASM, other

**ASM** – Antiseizure Medication, **AVM** – Ateriovenous Malformation, **AVUP**- Alert Verbal Unresponsive Pain, **BFMF** – Bimanual Fine Motor Function, **BP** – Blood Pressure, **CASCADE** – Childhood AIS Standardized Classification and Diagnostic Evaluation, CFCS – Communication Function Classification System, **GCS** – Glasgow Coma Scale, **GMFCS** – Gross Motor Function Classification System, **HR** – Heart Rate, **ICH** – Intracranial Haemorrhage, **IVH** – Interventricular Haemorrhage, **MACS** – Manual Ability Classification System, **mRS** – modified Rankin Scale, **OT** – Occupational therapy, **PedNIHSS** – Pediatric NIH Stroke Scale, **SAH** – Subarachnoidal Haemorrhage

2 **Data on Quality of Life and behavior**

The second main data source are questionnaires on quality of life and the Child Behaviour Checklist (CBCL), which are collected via mail. This will minimize loss to follow-up for this aspect of the study, as all patients and families will continue to be contacted even if they no longer attend or plan to attend medical appointments.

The KINDLR quality of life questionnaires by Ravens-Sieberer and Bullinger are a brief and flexible survey instrument that can be completed by children and adolescents as well as their parents [14–16]. The KINDLR questionnaires have been used in many studies and are characterized by high reliability, validity and user acceptance. They focus on the analysis of the following domains: physical well-being, emotional well-being, self-esteem, family, friends and school/education.

The CBCL is used to record behavioral problems, emotional abnormalities, somatic complaints and social skills of preschool-aged, school-aged children and adolescents from the parents’ perspective [17]. The survey covers the following sub-areas, known as problem scales Anxious/depressed, withdrawn/depressed, somatic complaints, social problems, thinking, (sleep) and repetition problems, attention problems, rule-breaking and aggressive behavior.

### Organization

The coordinating study center is located at the LMU University Hospital Munich and is responsible for the study protocol and the development of the CRFs, for the initiation and coordination of the study centres and the quality of the data entry. In addition, it collects quality of life data directly from families who have given their consent.

The database is located at the Institute for Medical Information Processing, Biometry, and Epidemiology (IBE) at the LMU Munich. Statistical analyses will also be performed there.

### Steering committee

A steering committee has been formed, consisting of national and international experts in the field of paediatric neurology, neuroradiology, haematology, rehabilitation medicine, intensive care medicine, and with representatives from Austria and the Netherlands to include country specific issues. The committee oversees the study, provides advice and decides on research proposals from investigators for data access.

### Ethics statement

The study has been approved by the ethics committee of the University of Munich (21–0571 and 3 amendments). Additionally, each local ethics committee has to approve the study for each study centre. PRiSMA is and will be conducted in accordance with the protocol, the Declaration of Helsinki and the principles of Good Clinical Practice.

PRiSMA is registried at the German Clinical Data Register (DRKS 00034715).

### Data management

Data are collected using a dedicated electronic data capturing system (REDCap). To comply with data protection laws, a hash value is generated from each patient’s insurance number and date of birth, which serves as a pseudonym. As the hashing procedure is non-reversible and only the pseudonym is saved, this system allows for combining a patients’ data through multiple institutions while ensuring that each centre has only access to its own data. Patients without insurance numbers will be assigned a pseudo-insurance number, which they can present at subsequent institutions.

Access to the data can be granted to interested scientists after sending in a proposal of the intended use. The decision on the proposal is made by the steering committee.

### Statistical analysis

The PRiSMA registry serves both to answer important questions and to generate new hypotheses exploratively.

Main outcome measurements are mortality, risk of recurrence, quality of life (measured via the KINDLR), behavioural and emotional function (measured via the CBCL) and neurological deficit and function (mRS, GMFCS, MACS, etc).

Continuous variables are described using the median and interquartile range or the mean and standard deviation. Categorical variables are described using relative and absolute frequencies. Event time data are described using Kaplan-Meier curves. In the presence of competing events, such as the occurrence of a recurrence and mortality without a prior recurrence, cumulative incidence curves considering both risks are used.

To identify prognostic factors, appropriate multiple regression models are conducted like linear, logistic, Cox proportional hazards or Fine and Gray model. If necessary, multiple imputation procedures for missing values have to be used.

Analyses will be performed with either SAS or R.

### Safety considerations

As the registry is purely observational, no safety issues arise.

### Current status

Start of recruitment was on 07/02/2024. Currently, 31 patients have been included in the registry (by 17 Nov 25). Up to now, 17 centres have signed the necessary documents and are eligible for patient enrolment, further study centres are being set up.

### Dissemination

Results of the study will be published in scientific journals and presented at national and international conferences. Key results will afterwards be shown on the study website (https://www.prisma-pediatricstroke.com/).

## Discussion

Our goal is to optimise diagnostics and personalise therapy in the acute and long-term phases to achieve the best possible outcomes for children and adolescents with stroke. To this end, we require a systematic database to develop appropriate diagnostic and therapeutic protocols. PRiSMA is a prospective multicentre registry for childhood stroke, recording data on acute care, long-term therapy, and health economics, while assessing the quality of life of affected children and adolescents over an extended period of time.

## Strengths

PRiSMA is the first stroke registry for children and adolescents in Germany, Austria and the Netherlands. With a German population of approximately 83 million people and additional centres in Austria and the Netherlands, it covers a large geographical area.

The registry includes children with both ischaemic and haemorrhagic stroke, the latter of which is more often characterised by severe functional limitations and higher economic costs [18]. Thanks to the PRiSMA structure, patients can be monitored over many years, and thanks to the construction of the pseudonymisation procedure developed specifically for this purpose allowing data linkage across sites, they can be monitored across many institutions in compliance with data protection regulations. Quality of life, behaviour and the impact of the stroke on future education are particularly important for children and adolescents and their families [19].

For this reason, PRiSMA has set up a special study to examine quality of life and behaviour. Data collected at regular intervals enables the needs of those affected to be identified directly and addressed more effectively. Acute stroke care has changed fundamentally in the last decade. Revascularisation procedures, such as thrombolysis and mechanical thrombectomy, have become standard practice in adult neurology and are now being used to treat children and adolescents with stroke [20–22].

However, this approach is not yet evidence-based. Therefore, PRiSMA’s ability to collect data during this era of revascularisation to assess impact on functional outcomes and collect data on complications in the acute phase of management and long-term outcomes is a significant advantage.

Thanks to the German Network Paediatric Stroke, which has been active and growing for over 10 years, the heterogeneity of the care landscape can be mapped in great detail. This is because the study includes not only university clinics, but also peripheral children’s clinics and paediatric rehabilitation clinics. Integrating the patient’s residence degree of urbanisation [23] enables potential inequalities in acute and long-term treatment for children and adolescents, depending on their location, to be identified. These criteria are an essential aspect of care research and, in the long term, could contribute to reducing inequalities and creating equal care opportunities for all children and adolescents, regardless of where they live, e.g., by facilitating access to telemedicine.

Furthermore, additional socio-economic factors, such as access to therapies, functional outcomes and quality of life, depending on parents’ level of education, can be identified. This highlights the need for an appropriate awareness campaign.

## Limitations

Currently, not all German children and adolescents who have had a stroke are covered by the registry. However, a nationwide network of >25 centres is being created across all federal states in Germany and this is set to expand with the initiation of more clinics including centres in Austria and the Netherlands.

From a statistical point of view, missing values are a very common issue in registries. To address this, we have developed a multifaceted strategy. Firstly, we have focused on including only items that are routinely collected by physicians treating paediatric stroke patients. Secondly, data will be curated throughout the study and missing values will be queried. Finally, multiple imputation techniques will be used to address any remaining missing data in the analysis.

Clearly, the estimation of the incidence of paediatric stroke can only be considered an underestimate, given that we do not yet know the coverage of our registry.

Another limitation is the lack of centralised review of imaging. This may result in missing or uncertain information regarding stroke aetiologies, particularly with regard to the classification of possible vasculopathies (e.g., focal cerebral arteriopathy – FCA vs. childhood primary angiitis of the CNS - cPACNS).

## Outlook

To minimise loss to follow-up, we are developing a minimal data set questionnaire, which will be sent to families alongside the QoL questionnaires. This will enable us to obtain basic information regarding therapy and diagnostics even if children and adolescents no longer have regular medical appointments or have moved to predominantly outpatient care, for which no entries are made in the register after some time.

Further satellite studies are planned, including investigations into the genetic predisposition to childhood stroke.

We also plan to capture data about spinal stroke via PRiSMA which are even rarer than cerebral infarcts in childhood and adolescence. They can cause permanent damage to the spinal cord and lead to long-term disabilities [24–26]. Currently, there are limited published data on spinal infarcts in this age group, and no systematic long-term surveys have been conducted.

## Amendments and termination of the project

Significant modifications to the project setup, protocol, and relevant project documents will be submitted to the responsible institutional review board for approval. The project does not have a predefined endpoint. However, PRiSMA may be terminated or suspended due to financial or personnel-related reasons.
